# Long-term clinical and radiological outcome of a cementless titanium-coated total knee arthroplasty system

**DOI:** 10.1007/s00402-023-05091-7

**Published:** 2023-10-13

**Authors:** Nina Hörlesberger, Maria Anna Smolle, Lukas Leitner, Georg Hauer, Andreas Leithner, Patrick Sadoghi

**Affiliations:** Department of Orthopaedics and Trauma, Medical University of Graz, Auenbruggerplatz 5, 8036 Graz, Austria

**Keywords:** ACS III, Outcome analysis, Tin-coated prosthesis, Total knee arthroplasty

## Abstract

**Introduction:**

To ensure a high-quality standard, it is important to frequently evaluate different prostheses models to avoid prostheses with high failure rates. Thus, the aim of the study was to evaluate the long-term outcome of the uncemented titanium-coated total knee arthroplasty (TKA) system (Advanced Coated System (ACS) III, Implantcast, GERMANY). We hypothesized that the ACS III would have a similar performance as other cemented TKA systems.

**Materials and methods:**

A total of 540 ACS III mobile-bearing knee joint prostheses were implanted in 495 patients. The visual analogue scale (VAS) score, Tegner activity score (TAS), knee society score (KSS), Western Ontario and McMaster (WOMAC) score, and the Short Form 12 (SF-12) score for the evaluation of quality of life (QoL) were taken after at least 9 years of follow-up. In addition, we measured range of motion (ROM) and assessed potential sex differences. In addition, the survival analysis was calculated at a median follow-up of 16.7 years.

**Results:**

At the final follow-up, 142 patients had died, and 38 had been lost to follow-up. The rate of revision-free implant survival at 16.7 years was 90.0% (95% CI 87.1–92.2%) and the rate of infection-free survival was 97.0% (IQR 95.2–98.2%). The reasons for revision surgery were aseptic loosening (32.9%), followed by infection (27.1%), inlay exchange (15.9%), and periprosthetic fractures (5.7%). At the clinical follow-up visit, the mean VAS score was 1.9 ± 1.9, the median TAS was 3 (IQR 2–4), and the mean KSS for pain and function were 83.5 ± 15.3 and 67.5 ± 25.2, respectively. The mean WOMAC score was 81.1 ± 14.9, and the median SF-12 scores for physical and mental health were 36.9 (IQR 29.8–45.1) and 55.8 (IQR 46.2–61.0), respectively. The mean knee flexion was 102.0° ± 15.4°. Male patients had better clinical outcome scores than female patients [SF-12 mental health score, *p* = 0.037; SF-12 physical health score, *p* = 0.032; KSS pain score (*p* < 0.001), and KSS functional score (*p* < 0.001)].

**Conclusion:**

The ACS III TKA system is a suitable option for the treatment of end-stage osteoarthritis of the knee joint because of its adequate long-term survival. Our findings are in line with published data on similar TKA systems that have shown favourable clinical scores in males.

**Level of evidence:**

Level III—Retrospective cohort study.

## Introduction

Since Gluck’s early work with ivory knee arthroplasty in 1890 and Shiers’ improved central core hinge principle in 1954 [1–3], the demands on total knee arthroplasty (TKA) evolved from simple osseous integration to long-term stability with maximum quality-of-life improvement. TKAs are being increasingly performed in Austria; the OECD country has one of the highest TKA implantation rates per inhabitant, marked by an increase of 13% from 2009 to 2015 [4]. In the US, a sevenfold increase in TKA is predicted by 2030 [5]. Implantation rates have continued to increase, because patients are living and staying active longer, thereby marking an increased demand for early rehabilitation and pain management after prosthesis implantation to achieve optimal longevity. In addition to patients’ expectations, the costs of prosthetic implants have increased in recent years and make up a high percentage of the total cost for TKA [6]. From the socioeconomic aspect, apart from the costs of the prostheses themselves [7], the increasing number of primary TKAs and consecutive revision surgeries will place a substantial burden on the total health care budget [8]. Apart from that, patients prefer to choose their prosthesis model and are willing to share the costs for more innovative technologies [7]. For this, several manufacturers produce more or less similar designs with various coatings to optimize patient outcomes.

The uncemented titanium-coated TKA system, Advanced Coated System (ACS) III (Implantcast, GERMANY), is a commonly used mobile-bearing prosthesis with a cobalt–chromium–molybdenum (CoCrMo) alloy that is known for its resistance to wear [9]. However, due to metal sensitivities, other coatings have been developed to counter the potential negative side effects of its ions on osteoblast-like cells and cytokines [10]. To lower the risk of negative side effects, a titanium nitride (TiN) coating has been developed. No adverse effects associated with TiN, a ceramic known for its scratch resistance, low friction coefficient, and reduced release of cobalt and chrome ions [9, 11, 12] have been reported in any randomized-controlled clinical trials investigating its benefits in terms of postoperative synovitis incidence, outcomes, and revision rate [9].

Although there are encouraging long-term results for TKA, with up to 80% of patients reporting improved function and less pain [13], some are still not fully satisfied [14], suggesting that more TKA models should be evaluated. Although the short-term outcomes of TiN-coated TKA systems have been reported, there is a lack of long-term evidence.

Therefore, the aim of this study was to investigate the uncemented titanium-coated total knee arthroplasty (TKA) system (Advanced Coated System (ACS) III, Implantcast, GERMANY) in a retrospective clinical and radiological study with a minimum clinical follow-up of 9 years and an implant survival analysis with a median follow-up of 16.7 years.

Our hypothesis was that this cementless ACS III would have a similar performance as other cemented systems in the literature with respect to clinical and radiological outcomes as well as survival and complication rates.

## Patients and methods

This study was approved by the local institutional review board (26-527 ex 13/14) and performed in line with the Declaration of Helsinki.

The inclusion criteria were patients with primary osteoarthritis who underwent cementless ACS III MB TKA between 2005 and 2006 at one single centre and who were followed up for a minimum of 9 years, at which time-point functional scores were obtained. In addition, follow-up on survival was performed at a median of 16.7 years using the database of the regional health system and phone interviews. Patients who underwent a secondary TKA, patients with pre-existing osteosynthesis of the distal femur or the proximal tibia, patients who underwent another knee surgery except for meniscus procedures, and patients with rheumatoid arthritis or an infection were excluded.

**Fig. 1:**
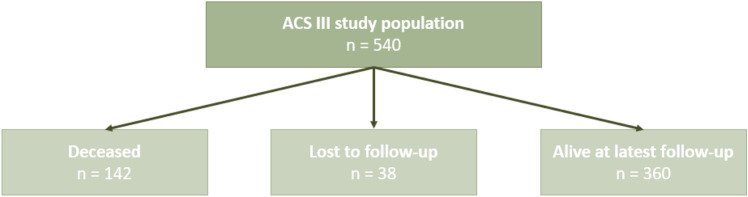
Flowchart

A total of 540 patients met the inclusion criteria for ACS III knee joint prosthesis surgery. At the final follow-up, 142 patients had died, and 38 had been lost to follow-up (Fig. 1).

The Advanced Coated System (ACS) III (Implantcast, Buxtehude, Germany) is a mobile-bearing bicompartmental knee prosthesis made of CoCrMo alloy coated with TiN. The deep-dish polyethylene inlay consists of ultrahigh-molecular-weight polyethylene (UHM-WPE).

Surgery was performed under general or spinal anaesthesia. A medial parapatellar approach with single-shot antibiosis with a cephalosporin or a lincosamide was used in all patients. All prostheses were implanted by or under the supervision of one out of three experienced senior surgeons using the mechanical aligned ligament balanced extension gap first technique. Postsurgical therapy featured full weight bearing and CPM (continuous passive motion).

The Visual Analogue Scale (VAS) score, Tegner Activity Score (TAS), Knee Society Score (KSS), Western Ontario and McMaster Universities Osteoarthritis Index (WOMAC) score, Short Form (SF)-12 score, and range of motion (ROM) were assessed preoperatively and at a minimum follow-up of 9 years. In addition, potential complications were analysed according to the Goslings and Gouma criteria [15].

The clinical scores were provided in German, the range of motion (ROM) was measured with a standard goniometer twice by two observers, and the inter- and intraclass correlations were good. Revision surgery was defined as the removal of at least removal one part of the prothesis.

In any patient with persisting pain or signs of infection, plain radiographs and CT scans were performed, and scintigraphy was performed if needed. In patients with suspected infection, joint aspiration and synovial analyses were performed. Diagnosis regarding infection was performed according to the EBJIS definition published by McNally et al. [16]. Painful arthroplasty was defined according to Djahani and Hofmann [17].

To ensure the independence of the study, patient data were evaluated at a different institution other than the hospital where the TKA was performed.

Stata Version 16.1 for Mac (StataCorp, College Station, Texas, US) was used for the data analysis. Means and medians were provided with corresponding standard deviations (SDs) and interquartile ranges (IQRs). Matched-pair Wilcoxon rank sum tests were performed for nonnormally distributed paired variables. Competing risk regression models were performed to assess overall complication-free survival, implant revision-free survival, and infection-free survival. A *p* value of < 0.05 was considered statistically significant.

## Results

Of the entire cohort, 354 patients were male (65.6%), and the mean age at surgery was 66.8 years ± 8.5 years. In 45 patients, bilateral TKA was performed. The median clinical follow-up was 9.8 years (IQR 9.0–10.4 years), and the median follow-up for survival was 16.2 years (IQR 10.6–16.8 years).

### Clinical outcome

Functional results were available for 401 patients at a median follow-up of 9.8 years (IQR 9.0–10.4 years). The mean Tegner score improved significantly from preoperative (3.0 ± 1.3) to postoperative (2.8 ± 1.2) with a *p* value < 0.001. The preoperative pain (VAS) score decreased significantly from 7.6 (± 1.8) to 1.9 (± 1.9) with a *p* value < 0.001.

The mean postoperative Knee Society Pain Score and postoperative Knee Society Functional Score were 83.5 (± 15.3) and 67.5 (± 25.2), respectively, and the mean WOMAC score was 81.8 points (± 14.9). The median SF-12 physical health score was 36.9 (IQR 29.8–45.1), and the median SF-12 mental health score was 55.8 (IQR 46.2–61.0). The mean ROM at the clinical follow-up was 102.0° (± 15.4°). Detailed outcome scores are illustrated in Table 1.

**Table 1:** Functional outcome scores at a minimum follow-up of 9 years (IQR 9.0–10.4 years) after total knee arthroplasty with the advanced coated system III

Score	Mean value	Standard deviation	Median	IQR
VAS	1.9	± 1.9		
Tegner AS			3	2–4
KSS pain	83.5	± 15.3		
KSS function	67.5	± 25.2		
WOMAC	81.8	± 14.9		
SF-12 PCS			36.9	29.8–45.1
SF-12 MCS			55.8	46.2–61.0
ROM-flexion	102.0	± 15.4		

### Sex differences

The clinical outcome scores were significantly different between the females and the males with regard to pre- and postoperative Tegner scores (*p* < 0.001). The preoperative VAS (*p* = 0.008) score and the VAS score at the latest clinical follow-up were significantly higher in the females than in the males (*p* = 0.002). In addition, the Knee Society Pain Score (*p* < 0.001) and the Knee Society Functional Score were significantly worse in the females than in the males (*p* < 0.001). In terms of the SF-12 mental health (*p* = 0.037) and physical health (*p* = 0.032) forms, the males scored higher than the females (Table 2).

**Table 2:** Functional outcome scores and sex differences at a minimum follow-up of 9 years (IQR: 9.0–10.4 years) after total knee arthroplasty with the advanced coated system III

	Female	Male	*p* value
Tegner Score Preoperative (mean ± SD)	2.8 ± 1.2	3.4 ± 1.4	< 0.001
Tegner Score Latest Clinical Follow-Up (mean ± SD)	2.6 ± 1.2	3.2 ± 1.2	< 0.001
VAS Preoperative (mean ± SD)	7.8 ± 1.3	7.4 ± 1.4	0.008
VAS Latest Clinical Follow-Up (mean ± SD)	2.1 ± 2.1	1.5 ± 1.5	0.002
KSS Pain (mean ± SD)	81.4 ± 16.6	87.8 ± 11.2	< 0.001
KSS Function (mean ± SD)	63.7 ± 25.6	75.4 ± 22.3	< 0.001
SF-12 Mental Health Part (median, IQR)	55.4 (43.4–60.8)	57.5 (47.7–62.1)	0.037
SF-12 Physical Health Part (median, IQR)	36.2 (28.9–44.3)	39 (31.4–46.8)	0.032

Pain and function scores, including the minimum and maximum values as well as the mean values and standard deviation 15 years after uncemented total knee arthroplasty (TKA), with sex differences

*VAS* Visual Analogue Scale, *Tegner AS* Tegner Activity Score, *KSS* Knee Society Score, *WOMAC* Western Ontario and McMaster Universities Osteoarthritis Index, *SF-12* Short form, *PCS* physical health part, *MCS* mental health part, *ROM-Flexion* Range of motion

### Complications

**Fig. 2:**
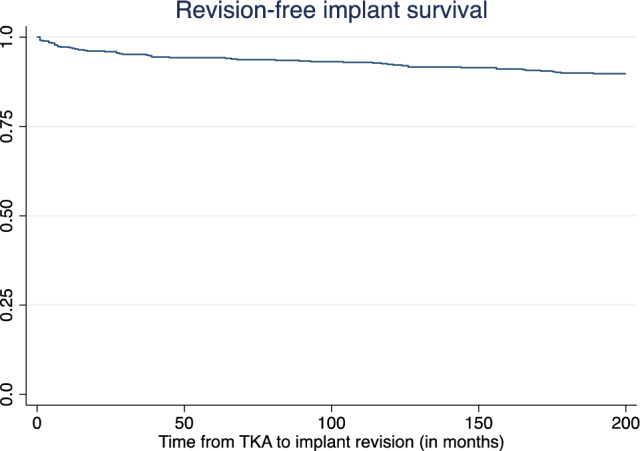
Revision-free implant survival for the entire cohort, as based on the competing-risk regression model with patient death as competing event

**Fig. 3:**
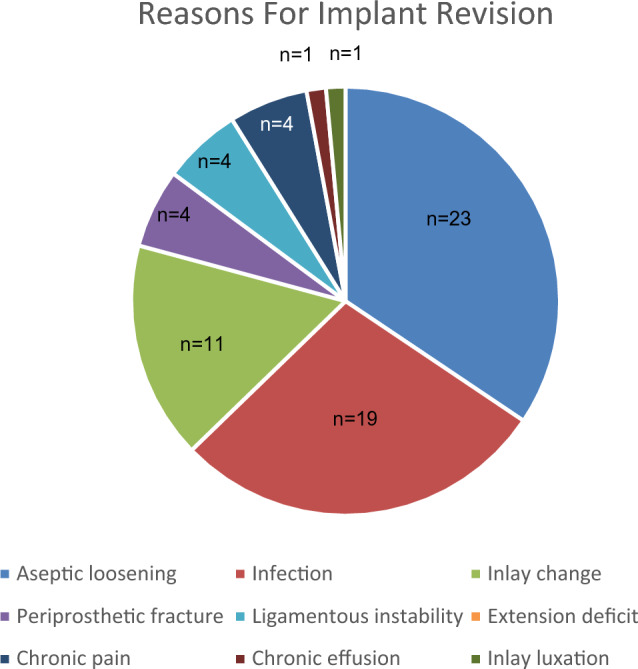
Reasons for implant revision after implantation of the advanced coated system III total knee arthroplasty

For the entire study population (540 ACS III TKAs, 495 patients), revision was required in 13.0% (*n* = 70) of patients at a median of 16.7 (IQR 10.5–17.4) years after ACS III TKA. There was no difference in the revision rate between the patients who died during the study period (14/142; 9.9%) and those who were still alive at the latest follow-up (56/342; 14.1%; *p* = 0.200). The rate of revision-free implant survival at 16.7 years was 90.0% (95% CI 87.1–92.2%; Fig. 2), and the rate of infection-free survival was 97.0% (IQR 95.2–98.2%). The most common reason for revision surgery was aseptic loosening (*n* = 23; 32.9%), followed by infection (*n* = 19; 27.1%), inlay exchange (*n* = 11; 15.7%), and periprosthetic fractures (*n* = 4; 5.7%). In 18.6% (*n* = 13) of patients who required revision surgery, the reasons other than those previously mentioned were evident (ligamentous instability (*n* = 4), extension deficit (*n* = 3), chronic pain without other detectable causes (*n* = 4), aseptic chronic reactive effusion (*n* = 1), and luxation of the inlay with consecutive changes in the inlay and tibial plateau (*n* = 1)). This is illustrated in Fig. 3.

## Discussion

The data support that the ACS III system provided excellent long-term outcomes and is well suited for the treatment of end-stage osteoarthritis of the knee joint when compared to other cemented systems. This is in line with a recent systematic review that reported that TiN-coated prostheses are not inferior to uncoated TKAs [18] and a mid-term follow-up matched-cohort study that reported satisfactory clinical outcomes and low revision rates for TiN-coated TKA [19]. Van Es et al. showed no difference in mid-term survival and function among cemented, uncemented, and hybrid fixation in mobile-bearing ACS TKA [20].

In 2014, Mont et al. reported a survival rate of 95.3% for cementless TKA in a review of 37 studies [21]. Prudhon et al. compared 200 cemented versus cementless TKAs in 2017 and described a survival rate of 90.2% for cemented TKAs at the 11-year follow-up [22]. Baier et al. found a 92.5% survival rate for the conventional TKA [23], while McCalden et al. found a 96.4% survival rate at the 15-year follow-up for 469 GENESIS II TKAs [24]. Carothers et al. compared rotating platform designs with meniscal bearing implants in mobile-bearing TKA and reported a 15-year survival rate of 96.4% vs. 86.5% [25]. Mohammed et al. assessed the outcome of titanium nitride mobile-bearing knee replacements and described a 15-year survival rate of 95.1% [26]. These studies are in line with the present findings, with clinically irrelevant differences in the survival rate, which was 90.0% in this study.

In total, the revision rate of the present study was 13.0% at a median of 16.7 years of follow-up. The main reasons for revision were aseptic loosening (32.9%), followed by infection (27.1%). This is in line with Sadoghi et al., who found aseptic loosening revision rates of 29.8% and infection rates of 14.8% in an analysis of worldwide arthroplasty registers in 2013 [27]. In other larger databases, the incidence of aseptic loosening after TKA ranges between 16 and 37%, while the incidence of infection ranges between 13 and 25% [28].

We found a significant reduction in pain scores of 5.7 points over a 10-year period postoperatively (*p* ≤ 0.001) as evaluated by the visual analogue score, which aligned with the primary goal of TKA and was superior that reported in the other studies on the ACS III system [9]. Furthermore, a significant reduction in the Tegner Activity Scale score was found from preoperative to latest clinical follow-up at a median of 9.8 years.

After comparing to our postoperative KSS Pain (83.5 points) and KSS Function (67.5) scores, mean postoperative WOMAC score (81.8), ROM (102.0°), as well as our median physical (36.9) and mental health (55.8) SF-12 scores, we found that the patients who underwent ACS III TKA had above-average scores in comparison to those reported in the other publications (Table 3). In a randomized-controlled trial with a small sample size (*n* = 68), Louwerens et al. reported good long-term results of TiN-coated cementless TKA but no better clinical outcome [29].

**Table 3:** Comparison of clinical and radiological outcomes of total knee arthroplasty (TKA) systems with our own data

Literature	Prosthesis	Mean follow-up (months)	*N*	VAS	KSS-P	KSS-F	WOMAC	SF-12 PCS	SF-12 MCS	ROM-flexion
Jauregui et al.[36]	Duracon© implant	132	145		84	73				
Kastner et al. [31]	LCS MB	168	138	1.28	72.5	74	82.5			100°
McCalden et al. [24]	Genesis II	192	238		85	61	69			
Mohammed et al. [26]	Previous version of ACS	79	222		77	69				101°
Van Hove et al. [9]	ACS MB Basic	60	51	~ 2	~ 90	~ 69				116°
Van Hove et al. [9]	LCS MB	60	50	~ 2	~ 90	~ 70				111°
Vielgut et al. [37]	LCS MB	178.8	260	1.42			> 80			
Vogt et al. [38]	LCS MB	136.8	52		78	66		37	47	105.8°
Yang et al. [39]	NextGen CR	120	113	1.15			87.6			131.8°
Own results	ACS III		540	1.9	83.5	67.5	81.8	36.9	55.8	102.0°

These evaluated different TKA systems by usual scores such as *VAS* Visual Analogue Scale, *KSS-P* Knee Society Score Pain, *KSS-F* Knee Society Score Function, *SF-12* Short Form 12, *PCS* physical health part, *MCS* mental health part, *ROM-Flexion* Range of motion—flexion

Furthermore, females had higher initial and latest follow-up VAS scores than males, as well as poorer KSS function and pain scores and worse SF-12 physical and mental health scores. Present sex-specific significant outcome differences are in accordance with O’Connor’s results, who reported less pain and better function in males in 2011 [30], and with Kastner et al., who showed higher VAS scores in females [31].

Long-term outcome studies are relevant to present trustworthy data for both surgeons and patients. This is the first study to describe the long-term clinical outcome of ACS III TKA. Such studies have been published in the highest ranked orthopaedic journals on a regular basis [32]. Nevertheless, we want to report that the retrospective, uncontrolled design is the first limitation of this study. Another limitation is the high rate of deceased patients at time of analysis. In addition, functional outcome depends not only on preoperative conditions and the surgical setting but also on physiotherapy and other factors that were not considered. BMI, which has an impact on complications and outcome, was not clearly monitored in this study [33, 34]. It must be mentioned that the Knee Society Pain and Functional Scores as well as the WOMAC score are missing. However, it must be emphasized that the results were evaluated outside of the primary institution at which the surgery was performed to avoid observer bias and to maintain strict impartiality [35]. Furthermore, it must be mentioned that survival rates were evaluated electronically, as there is a regional health database where all regional revision centres are included.

## Conclusion

We present the first study that evaluates the long-term outcome of the ACS III system over a minimum follow-up of 9 years and an implant survival analysis with a median follow-up of 16.7 years. The present findings are in line with published data on similar TKA systems. Favourable clinical scores are evident in male patients. We believe that the ACS III TKA system is a suitable option for the treatment of end-stage osteoarthritis of the knee joint.

## Data Availability

Data is available upon reasonable request from the corresponding author.
